# Differences in lower extremity kinematics and kinetics during a side-cutting task in patients with and without chronic ankle instability

**DOI:** 10.3389/fspor.2025.1593231

**Published:** 2025-06-06

**Authors:** Yue Xu, Yizhang Mo, Chengxiao Liu, Haofan Chen, Jianhao Zhao, Wanxia Zhang, Yang Yu, Yulin Li, Lina Wang, Yingge Yuan, Zhongyue Sun, Guoxin Ni, Bin Song

**Affiliations:** ^1^ Department of Joint Surgery and Sports Medicine, The Sixth Affiliated Hospital, Sun Yat-sen University, Guangzhou, China; ^2^ Biomedical Innovation Center, The Sixth Affiliated Hospital, Sun Yat-sen University, Guangzhou, China; ^3^ School of Sports, Guangxi College of Sports Education, Nanning, China; ^4^ Sports Kinesiology Department, Anhui Professional & Technical Institute of Athletics, Hefei, China; ^5^ College of Sports Medicine and Rehabilitation, Beijing Sport University, Peking, China; ^6^ Cardiac Surgery, Peking University International Hospital, Peking, China; ^7^ Department of Rehabilitation Medicine, The First Affiliated Hospital of Xiamen University, Xiamen, China; ^8^ Department of Traditional Chinese Medicine and Rehabilitation, Henan Vocational College of Nursing, Anyang, China

**Keywords:** CAI, side-cutting, kinematics, kinetics, lower extremity, joint biomechanics

## Abstract

**Background:**

Patients with chronic ankle instability (CAI) have demonstrated altered hip and knee movement strategies during walking and running, but these movement modalities do not involve changes in speed and direction, making it difficult to simulate the conditions of real sports, whereas side-cutting task can provide CAI patients with a more realistic athletic challenge. However, there is limited literature examining the kinematic and kinetic differences in the hip, knee, and ankle joints of CAI patients during the side-cutting task.

**Objective:**

To assess differences in lower extremity joint kinematics and kinetics during the side-cutting task in individuals with and without CAI.

**Design:**

Cross-sectional study.

**Participants:**

48 males, 24 in each of the CAI group and healthy control group; 40 females, 20 in each of the CAI group and healthy control group.

**Methods:**

Lower extremity three-dimensional kinematic and kinetics data were evaluated by using a three-dimensional motion analysis system during the initial contact (IC) and toe off (TO) while side-cutting.

**Results:**

Compared with healthy controls, male patients with CAI exhibited greater hip flexion and external rotation angles, knee internal rotation angles, smaller knee flexion angles and ankle inversion angles, greater hip external rotation moments, and greater knee abduction moments; female patients with CAI exhibited smaller hip and knee flexion angles, greater hip external rotation angles, larger ankle inversion angles and internal rotation angles, smaller hip external rotation moments, and greater knee abduction moments.

**Conclusion:**

Our findings indicate that patients with CAI exhibit altered lower limb joint kinematics and kinetics during side-cutting task, with significant sex-specific differences. These movement pattern changes involve proximal joint compensation to stabilize the unstable distal ankle joint; however, these compensatory changes are not always favorable. The greater hip external rotation moment and greater knee internal rotation angle demonstrated by male CAI patients, the smaller hip flexion angle and greater ankle internal rotation angle demonstrated by female CAI patients, and the smaller knee flexion angle and greater knee abduction moment common to both sexes may impair the lower limb's ability to effectively absorb and dissipate ground reaction forces, potentially elevating the risk of lower extremity injuries.

## 1 Introduction

Lateral ankle sprains represent the most prevalent musculoskeletal injury in athletic populations, particularly in sports demanding rapid directional changes such as basketball, soccer, and volleyball (1). Despite high incidence rates, epidemiological data reveal that fewer than 50% of affected athletes pursue professional treatment post-initial injury (2). This clinical apathy contributes to the development of chronic ankle instability (CAI) in approximately 40% (3) of cases—a pathological condition characterized by recurrent sprains, persistent pain, persistent swelling, functional limitations, and self-reported functional impairment persisting beyond 12 months from initial injury (4). Of particular concern is the progressive articular degeneration associated with CAI, with 68%–78% of patients eventually developing ankle osteoarthritis (5), significantly compromising both athletic performance and quality of life (6).

Lateral ankle sprains primarily arise from excessive subtalar inversion, internal rotation, and talocrural plantar flexion of the ankle complex (7, 8). As a critical node in the lower extremity kinetic chain, ankle dysfunction inevitably propagates mechanical alterations to proximal joints (9). Contemporary research confirms distinct kinematic and kinetic deviations in CAI patients during basic ambulatory tasks. Specifically, during walking, CAI patients exhibit increased ankle inversion angles (10) and moments (11), decreased knee external rotation angles (12), and elevated knee abduction moments (11); during running, they demonstrate increased plantarflexion and inversion angles compared to healthy individuals (10, 13). However, these observations derive predominantly from steady-state locomotion analyses, which inadequately replicate the dynamic demands of sport-specific maneuvers.

The side-cutting task, prevalent in many sports, imposes complex biomechanical demands (14, 15)—including velocity modulation, directional changes, and impact loading—that exacerbate functional challenges for individuals with CAI. Three-dimensional motion analyses demonstrate altered lower extremity kinematics and kinetics during this task, though current evidence remains contradictory (16). While consensus exists regarding increased hip flexion in CAI populations (17–19), substantial discrepancies persist across studies examining multiplanar hip movements and distal joint mechanics (17–19). Methodological heterogeneity in task protocols, footwear standardization, and sex distribution likely contributes to these inconsistencies—a critical consideration given established sex-based differences in neuromuscular control (20) and movement strategies (21, 22).

Emerging kinetic analyses reveal compensatory patterns including reduced ankle eversion moments and modified proximal joint loading (17). Jeffrey et al. (23) identified diminished knee adduction and ankle eversion moments coupled with elevated plantarflexion moments, while Kim et al. (19) proposed that increased hip/knee flexion represents a kinetic adaptation to mitigate ground reaction forces. These proximal compensations, potentially serving dual roles in functional adaptation and injury risk potentiation (17), underscore the need for comprehensive biomechanical profiling across the kinetic chain.

The primary aim of this study was to address critical knowledge gaps through sex-stratified analysis of lower extremity biomechanics during standardized side-cutting task. We hypothesize that patients with CAI at the amateur sports level will demonstrate distinct kinematic and kinetic signatures compared to healthy controls, particularly manifesting as altered hip flexion patterns, modified knee joint loading mechanics, and compensatory ankle moment adaptations. Elucidating these movement strategies holds dual clinical significance: informing targeted rehabilitation protocols for ankle instability management while advancing mechanistic understanding of CAI progression.

## 2 Methods

### 2.1 Participants

The sample size was calculated using the two-sample mean comparison method with data from Lin et al. (24). Based on this data analyzed through PASS 11.0 software (NCSS, USA), a minimum of 9 subjects per group would provide 90% statistical power to detect mean differences at the 0.05 significance level. Accounting for an anticipated 10% dropout rate, the adjusted sample size required is at least 10 subjects per group. A total of 88 individuals, 48 males and 40 females, who met the criteria were enrolled in the study, which was approved by the Ethics Committee (2021036H), and all subjects gave informed consent and signed the informed consent form.

Inclusion and exclusion criteria: This study referred to the screening criteria for chronic ankle instability proposed by The International Ankle Consortium (25). The criteria were as follows:

(1) CAI group inclusion criteria:
  a. age 18–30 years;
  b. At least one significant right lateral ankle sprain requiring protective weight bearing and/or immobilization;
  c. History of two or more lateral ankle sprains at the right ankle joint;
  d. Multiple episodes of right ankle instability and/or “giving way”;
  e. Cumberland Ankle Instability Tool (CAIT) ≤ 24 and The Identification of Functional ankle instability (IdFAI) ≥ 11;
  f. Physical activity (running, football, basketball, and other general sports) for at least 30 min at least 3 times per week;
  g. No specialized training is required, and the Tegner score ≤ 5.
(2) Inclusion criteria for healthy control group:
  a. age 18–30 years;
  b. No history of lateral ankle sprain;
  c. Physical activity (running, football, basketball, and other general sports) for at least 30 min at least 3 times per week;
  d. No specialized training is required, and the Tegner score ≤ 5.
  e. Cumberland Ankle Instability Tool (CAIT) > 24 and The Identification of Functional ankle instability (IdFAI) < 11;
  f. WFQ-R > 0.
(3) Exclusion criteria:
  a. acute ankle sprain;
  b. previous history of lower extremity fracture, surgery, and major musculoskeletal injury (except history of lateral ankle sprain in the CAI group);
  c. Pain or swelling of the ankle joint at the time of the test;
  d. Positive anterior talar drawer test and talar tilt test;
  e. Knee injuries (anterior cruciate ligament injury, posterior cruciate ligament injury, meniscus injury, articular cartilage injury, patellar dislocation, medial collateral ligament injury, lateral collateral ligament injury, intra-articular fracture, patellar tendon tear, etc.);
  f. Beighton score > 4;
  g. Other interventional clinical treatments or trials within 3 months.

### 2.2 Instrumentation

#### 2.2.1 Questionnaires and scales

Cumberland Ankle Instability Tool (CAIT) (26): This questionnaire investigate the subjective sensation of the ankle joint in patients with CAI during different types of daily activities. A total score of 30 points was used, with lower scores indicating poorer ankle stability, and scores of less than 24 points were considered to indicate chronic ankle instability (27).

The Identification of Functional instability (IdFAI) (28): This questionnaire investigate the injury and frequency of perceived instability in patients with CAI. A total score of 37 points was used, with higher scores representing poorer ankle stability, and scores greater than or equal to 11 points were considered to indicate the presence of CAI (28).

The Waterloo Footedness Questionnaire-Revised (WFQ-R) (29): This scale investigate which foot the subject would use in different situations. Scores range from −20–20, with negative values indicating the left side as the dominant side and positive values indicating the right side as the dominant side.

The Tegner activity score (30): This scale commonly used to assess the level of exercise, with 10 levels, ranging from 1–10 in descending order. A score of less than or equal to 5 is considered to be an amateur level.

#### 2.2.2 Motion capture systems and force plates

Kinematic data from 16 infrared reflective markers attached to the subject's body were acquired using an 8-camera infrared high-speed motion capture system (Motion Analysis Raptor-4, USA) and Cortex software (Motion Analysis Corp, Santa Rosa, CA, USA) at a sampling rate of 200 Hz. Ground reaction force data were acquired using two 3D force plates (Kistler 9281CA, Switzerland) at a sampling frequency of 1,000 Hz during the movement. The motion capture system and the force plates were synchronized using Cortex software.

### 2.3 The side-cutting task

**Figure 1:**
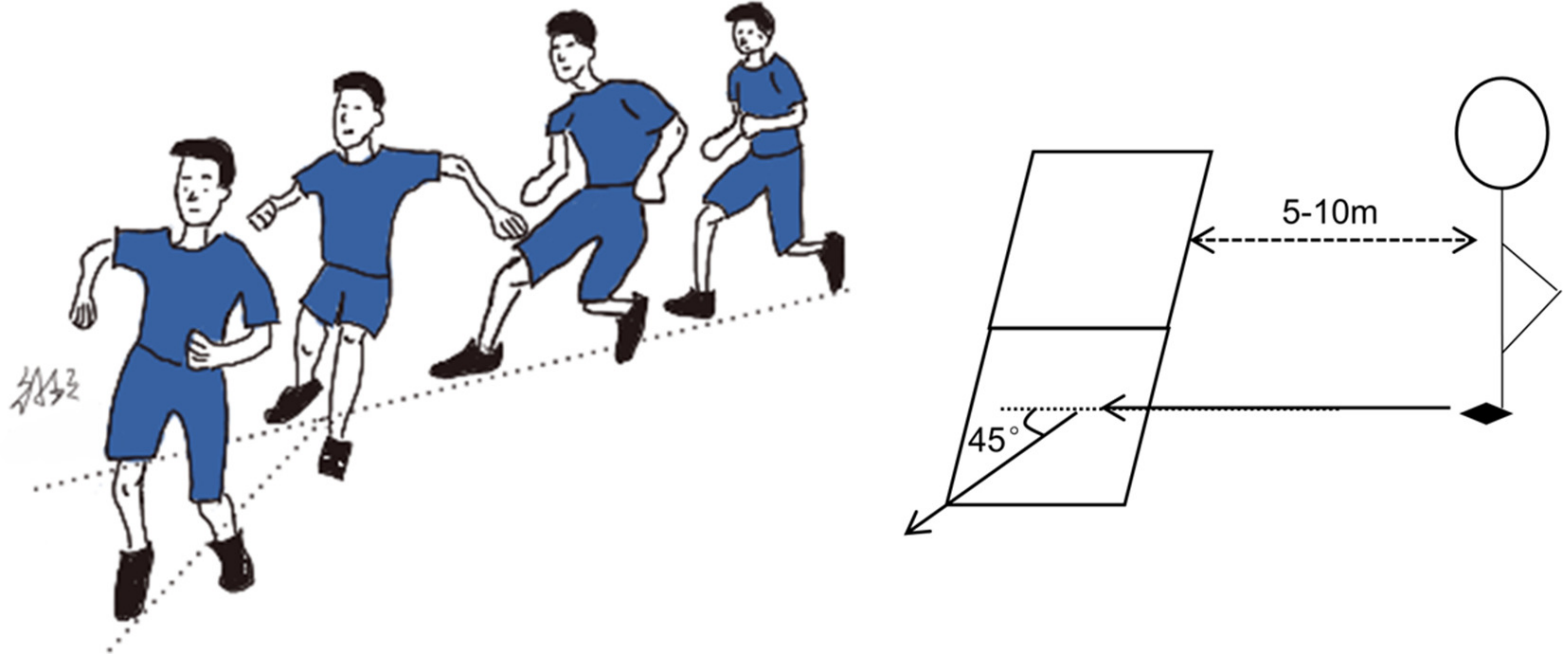
Schematic diagram of the side-cutting task; the subject is asked to start running with full power 5–10 m away from the force plate, using the affected or dominant side as support on the force plate, and then to change direction sharply in the original direction of motion by 45° to the left front (right side support), and to continue to run at full speed for 4–5 paces and then stop with a cushion to end the maneuver.

The side-cutting task: Subjects were asked to start running at full power 5–10 m from the force plate, with the affected or dominant side supported on the table, and then to change direction sharply by 45° to the left anterior (right support) in the original direction of motion and to continue running at full speed for 4–5 paces to a cushioned stop (Figure 1), ending the maneuver (31).

### 2.4 Procedures

In this study, the Cumberland Ankle Instability Tool (CAIT), The Identification of Functional ankle instability (IdFAI) and The Tegner activity score were used to screen and group the subjects and to investigate their ankle function. According to the inclusion criteria, the subjects were divided into the CAI group and the healthy control group. The Waterloo Footedness Questionnaire-Revised (WFQ-R) was used to determine the dominant side of the subjects.

In order to eliminate the influence of different sports equipment (footwear and clothing) on the biomechanical data, the subjects changed into uniform sports socks and shoes, leggings, in addition to sports underwear for female subjects before the warm-up started.

After changing shoes, socks, and clothing, the subject followed the trainer for a 5 min warm-up session, which was based on the Harmony Knee Prevention Training Program by Kiani et al. (32) and consisted of jogging, backward running, high leg lifts, and zigzag running. It ensured that the subjects were fully mobilized and activated before the start of the test.

After the warm-up, in order to ensure that the subjects' familiarity with the side-cutting movement was basically the same, the subjects were required to follow the trainer to learn the movement, and needed to successfully complete the movements 3 times under the supervision of the trainer to be regarded as the completion of the study. The CAI patients' test side was the affected side (right side), and the healthy control groups' test side was the dominant side (right side).

After making sure that the subjects had mastered the test movements, the experimenter pasted reflective markers for the subjects, which were set according to the Halen-Hayes model of the lower limb with a total of 16 points (33), and were pasted on the subjects' bilateral acromion, right scapula point, bilateral anterior supraspinous iliac spines, bilateral greater trochanters of the femur, and medial femoral condyles on the affected or dominant side, lateral condyles of femur, tibial tuberosity, medial malleolus of the tibia, lateral malleolus of the fibula, first metatarsal trochanter, fifth metatarsal trochanter, heel point, and midpoint of posterior supraspinous iliac spine, respectively.

In order to avoid fatigue, there is a 1 min break between each test maneuver, and each subject is required to collect valid data 3 times for the test maneuver, and the quality of the test maneuver is supervised by the trainer. If the foot does not make contact with the force plate during the movement or any reflective marking point is lost during the movement, the failure of the test will not be counted as the number of valid completions. Finally, calculate the average of the three datasets as the final result.

### 2.5 Data processing

Lower extremity hip, knee, and ankle kinematic and kinetic data were processed using Cortex analysis software (Motion Analysis Corp, SantaRosa, CA, USA).

The threshold value of the force plates was set to 20 N, and the moment when the vertical ground reaction force (GRF) was greater than 20 N for the first time during the maneuver was defined as the moment of initial contact (IC), and the moment when the vertical GRF was less than 20 N for the first time was defined as the moment of toe off (TO). The period from IC to TO was defined as the stance phase (SP) and normalized to 100% of the time.

Raw 3D coordinate data and GRF data for all reflective marker points were filtered through a fourth-order low-pass Butterworth filter with a cutoff frequency of 13.3 Hz. Lower extremity models (including foot, calf, thigh, and pelvic segments) were created based on the Helen-Hayes model using Cortex analysis software, and the rigid body model in the software was corrected by the height and weight of the subject.

The Euler angle method was used to calculate the three-dimensional angles of the hip, knee and ankle joints, in which the hip angle was defined as the Euler angle between the pelvic coordinate system and the thigh coordinate system. The first rotation was around the *Y*-axis to obtain the angles of flexion and extension (+ for extension,−for flexion), the second rotation was around the *X*-axis to obtain the angles of adduction and abduction (+ for abduction,−for adduction), and the third rotation was around the Z-axis to obtain the angles of internal and external rotation (+ for internal rotation,−for external rotation). The knee angle is defined as the Euler angle between the thigh coordinate system and the calf coordinate system, with the *Y*-axis obtaining the flexion and extension angles (+ for flexion,−for extension), the *X*-axis obtaining the adduction and abduction angles (+ for abduction and−for adduction), and the Z-axis obtaining the internal and external rotation angles (+ for internal rotation and−for external rotation). The ankle angle is defined as the Euler angle between the calf and ankle coordinate systems. The *Y*-axis obtains the angle of plantar and dorsiflexion (+ for plantarflexion,−for dorsiflexion), the *X*-axis obtains the angle of inversion and eversion (+ for eversion,−for inversion), and the Z-axis obtains the angle of internal and external rotation (+ for internal rotation,−for external rotation). The net joint moments of the lower extremity at the hip, knee, and ankle were obtained based on the calculation of inverse kinetics and normalized by dividing by the subject's body weight.

### 2.6 Statistical analysis

Microsoft Excel 2016 (Microsoft Corp., USA) was used to organize and calculate the data. IBM SPSS 22.0 statistical software (SPSS Corp., USA) and Python 3.10 software (The Python Software Foundation, http://www.python.org) were applied to analyze the data. Continuous variables were expressed as $\bar{x}\pm s$, normality was tested using the Shapiro-wilk test, and demographic data were compared at baseline using the independent samples *t*-test or the Mann–Whitney U rank sum test. The distribution of kinematic and kinetic data was assessed for normality using the D' Agostino-Pearson test. A one-dimensional non-parametric permutation method (SnPM) through the spm1d 0.4 analysis package (https://www.spm1d.org) (12, 34) was employed to compare hip, knee, and ankle joint kinematic and kinetic curve data between the CAI group and the healthy control group from IC (1%) to TO (100%), utilizing either the two-independent-sample *t*-test or non-parametric rank-sum test as appropriate.The significance threshold for all analyses was fixed at *α* = 0.05, and the highest Cohen's d effect size was calculated when a statistically significant difference between groups was observed, with effect sizes assessed as weak (0.2 < *d* < 0.49), moderate (0.50 < *d* < 0.79), and large (*d* > 0.80) (35). Statistical plots were drawn by Python 3.10 software and Microsoft PowerPoint 2016 (Microsoft Corp., USA) software.

## 3 Results

### 3.1 General characteristics of the subjects

A total of 88 subjects who met the inclusion criteria were enrolled in this study, including 48 males, 24 in each of the CAI group and healthy control group, and 40 females, 20 in each group. There were no statistically significant differences in CAIT scores and IdFAI scores between sexes, and there were no statistically significant differences in age, height, weight, and body mass index (BMI) between groups within each sex (*p* > 0.05), and the groups were comparable at baseline (Table 1).

**Table 1:** General characteristics of the subjects $\bar{x}\pm s$.

Sex	CAI Group		Healthy control group	
	Male	Female	Male	Female
Sample Size (*n*)	24	20	24	20
Age (y)	21.83 ± 2.90	22.65 ± 3.62	22.08 ± 3.28	22.70 ± 3.69
Height (m)	1.80 ± 0.06	1.65 ± 0.08	1.78 ± 0.04	1.65 ± 0.07
Mass (kg)	78.45 ± 10.91	59.58 ± 7.89	74.96 ± 7.52	56.90 ± 16.48
BMI (kg/m^2^)	24.11 ± 2.72	21.78 ± 2.07	23.50 ± 1.74	21.67 ± 2.18
CAIT Score	18.21 ± 2.32	18.30 ± 3.28	29.26 ± 1.44	29.33 ± 1.32
IdFAI Score	17.67 ± 4.60	17.45 ± 3.98	6.84 ± 3.65	7.14 ± 3.41
Tegner Score	3.63 ± 0.65	3.35 ± 0.67	3.50 ± 0.67	3.19 ± 0.40

BMI, body mass index.

### 3.2 Alterations in lower limb joint kinematics during side-cutting task

**Figure 2:**
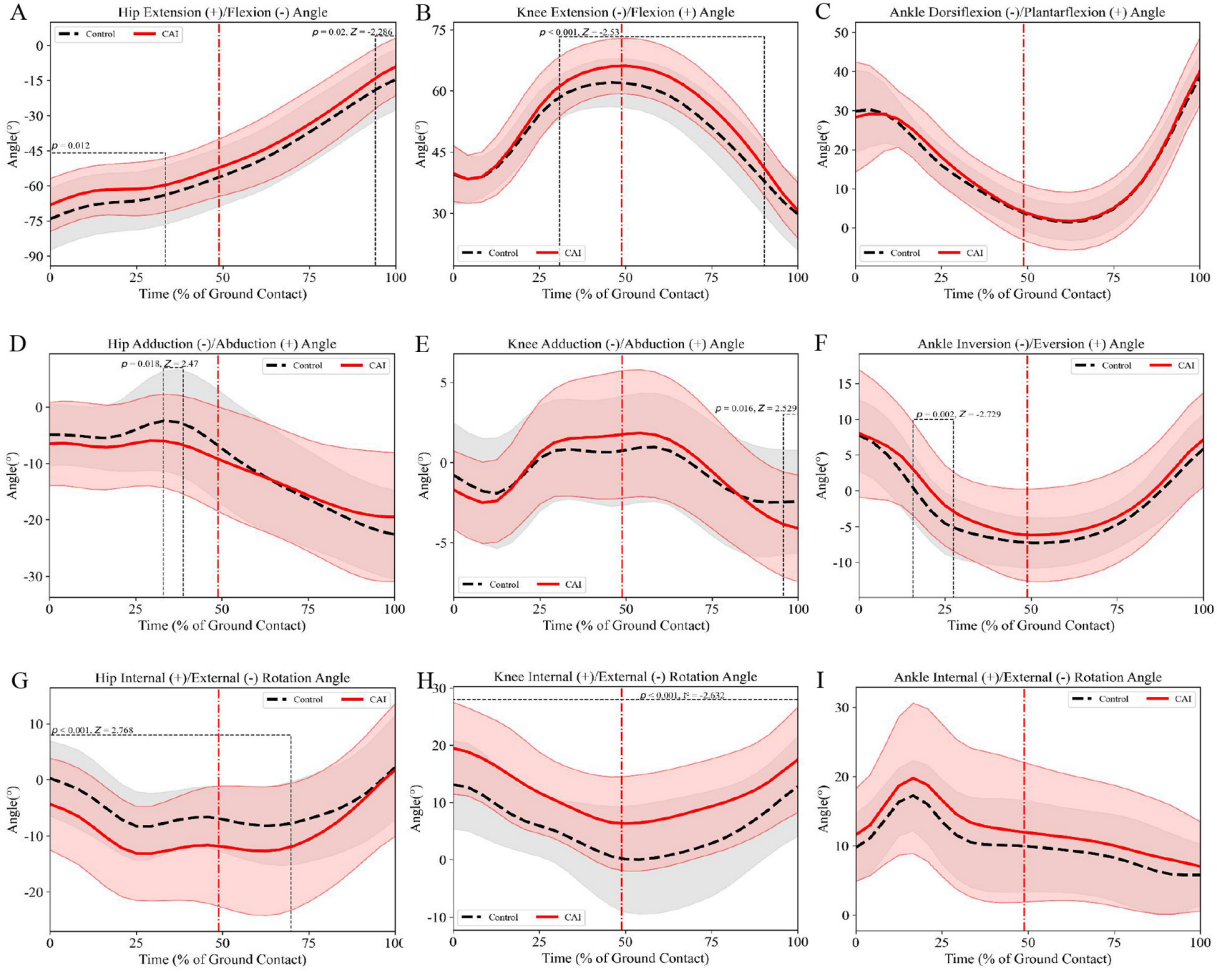
Male lower limb joint angles (mean ± SD) during side-cutting task: **(A–C)** sagittal plane angles, **(D–F)** frontal plane angles, **(G–I)** transverse plane angles. The red dotted line indicates the moment of maximum knee flexion during the maneuver.

The sagittal plane results demonstrated that compared to the control group, male patients with CAI exhibited: (a) greater hip flexion angles during 0%–28% of the stance phase (mean difference = 5.71°, *p* = 0.012, *d* = 0.48; Figure 2A) and 92%–100% of the stance phase (mean difference = 5.13°, *p* = 0.02, *d* = 0.40; Figure 2A); (b) reduced knee flexion angles during 28%–88% of the stance phase (mean difference = 4.14°, *p* < 0.001, *d* = 0.61; Figure 2B). In the frontal plane, male patients with CAI showed decreased ankle inversion angles during 16%–28% of the stance phase (mean difference = 2.67°, *p* = 0.002, *d* = 0.51; Figure 2F). Transverse plane analyses revealed that male patients with CAI displayed: (a) increased hip external rotation angles during 0%–64% of the stance phase (mean difference = 4.88°, *p* < 0.001, *d* = 0.61; Figure 2G); (b) greater knee internal rotation angles throughout the stance phase (mean difference = 5.99°, *p* < 0.001, *d* = 0.76; Figure 2H).

**Figure 3:**
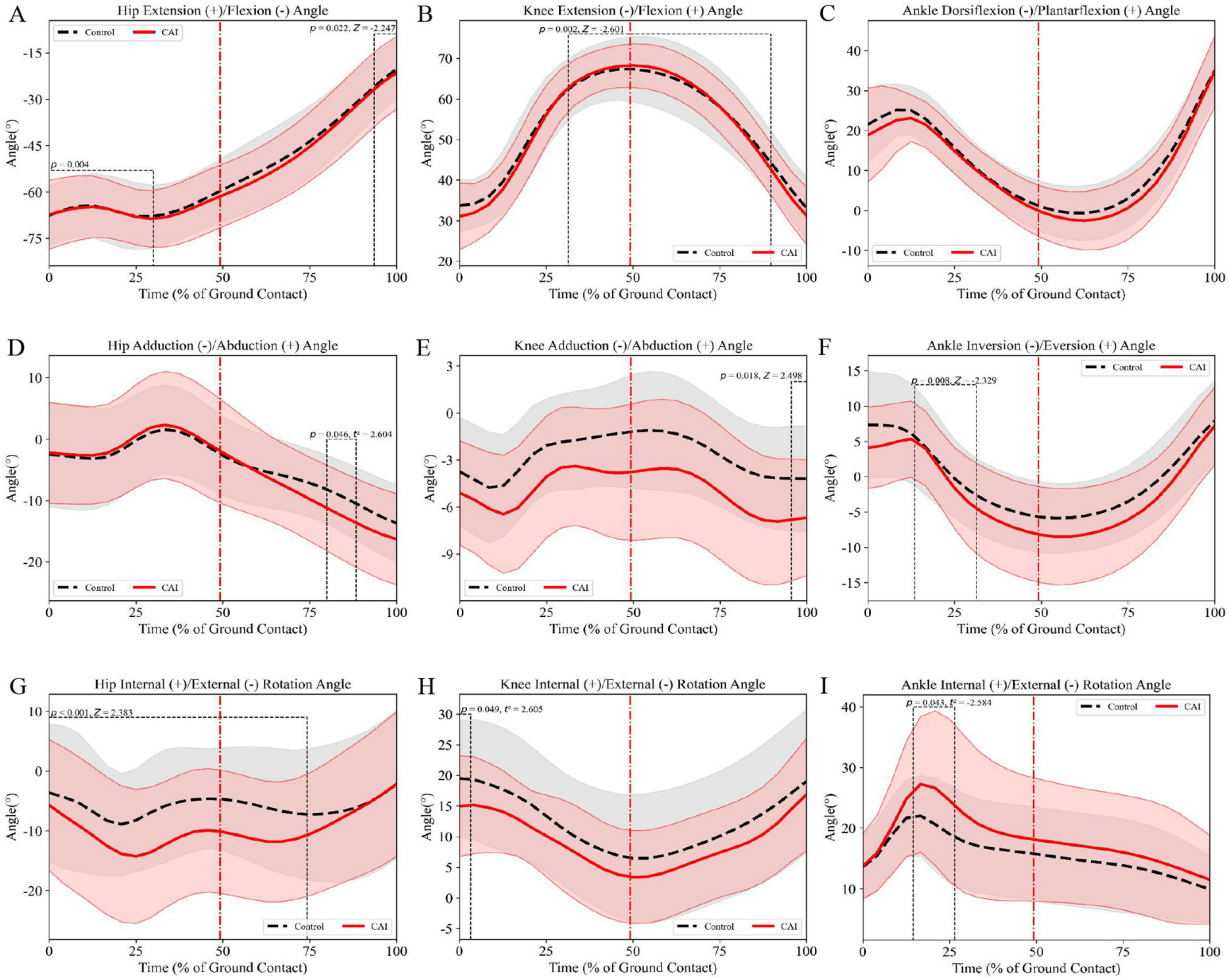
Female lower limb joint angles (mean ± SD) during side-cutting task: **(A–C)** sagittal plane angles, **(D–F)** frontal plane angles, **(G–I)** transverse plane angles. The red dotted line indicates the moment of maximum knee flexion during the maneuver.

The results of sagittal plane angles revealed that compared with the control group, female patients with CAI exhibited: (a) significantly smaller hip flexion angles during 0%–28% (mean difference = 0.14°, *p* = 0.004, *d* = 0.01; Figure 3A) and 92%–100% (mean difference = 0.90°, *p* = 0.022, *d* = 0.08; Figure 3A) of the stance phase; (b) reduced knee flexion angles during 32%–88% of the stance phase (mean difference = 0.38°, *p* = 0.002, *d* = 0.05; Figure 3B). In the frontal plane, female patients with CAI demonstrated greater ankle inversion angles during 12%–32% of the stance phase (mean difference = 0.97°, *p* = 0.008, *d* = 0.16; Figure 3F). Transverse plane analysis showed that female patients with CAI presented: (a) increased hip external rotation angles during 0%–72% of the stance phase (mean difference = 5.00°, *p* < 0.001, *d* = 0.49; Figure 3G); (b) greater ankle internal rotation angles during 12%–24% of the stance phase (mean difference = 4.77°, *p* = 0.043, *d* = 0.51; Figure 3I).

### 3.3 Alterations in lower limb joint kinetic during side-cutting task

**Figure 4:**
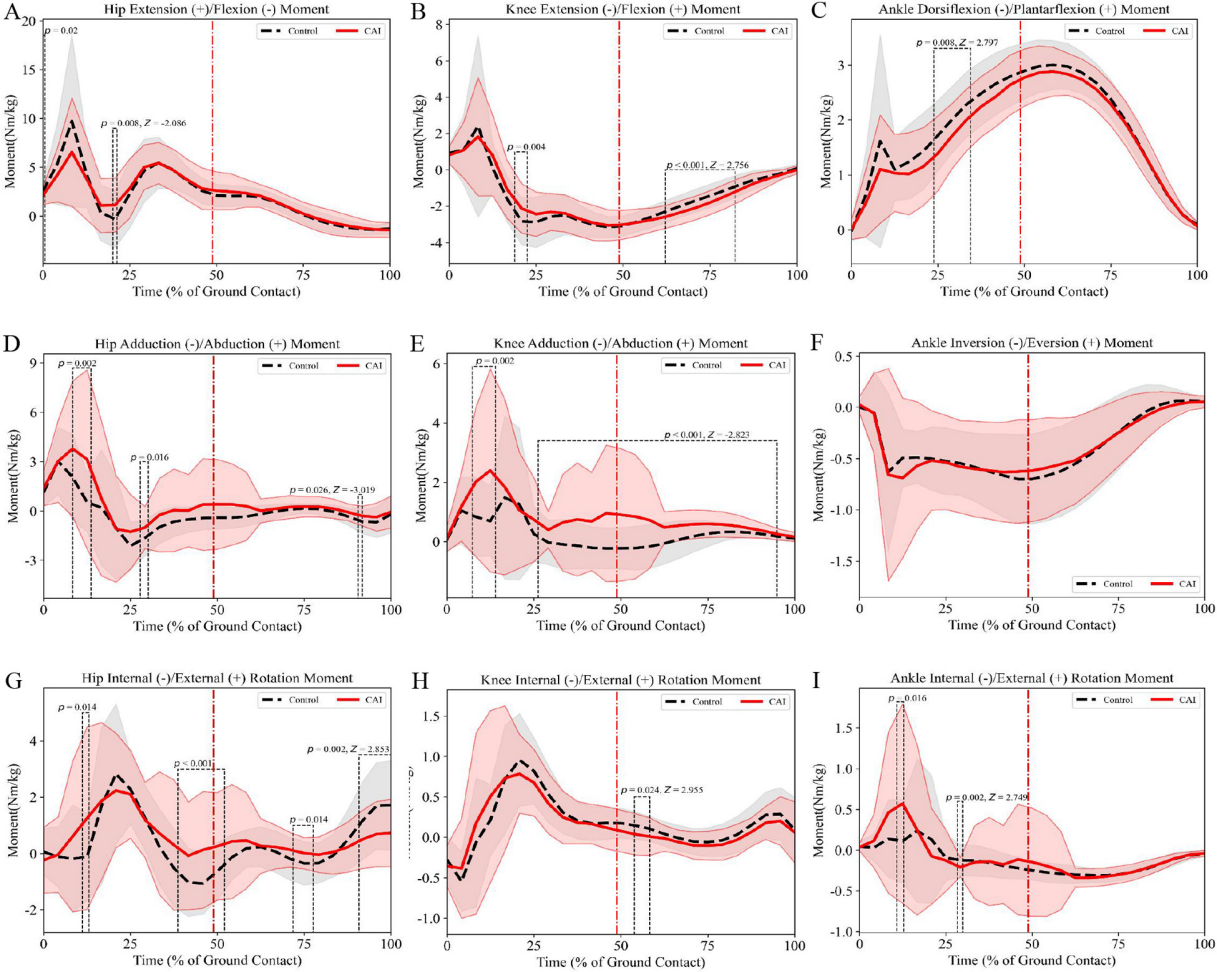
Male lower limb joint moments (mean ± SD) during side-cutting task: **(A–C)** sagittal plane moments, **(D–F)** frontal plane moments, **(G–I)** transverse plane moments. The red dotted line indicates the moment of maximum knee flexion during the maneuver.

Compared with the control group, frontal plane moment analysis revealed that male patients with CAI exhibited significantly greater knee abduction moments during 8%–12% (mean difference = 1.18 Nm/kg, *p* = 0.002, *d* = 0.63; Figure 4E) and 24%–92% (mean difference = 0.59 Nm/kg, *p* < 0.001, *d* = 0.67; Figure 4E) of the stance phase. Transverse plane moment analysis demonstrated that male patients with CAI: (a) displayed increased hip external rotation moments at 8%–12% (mean difference = 0.79 Nm/kg, *p* = 0.014, *d* = 0.34; Figure 4G), 36%–48% (mean difference = 0.99 Nm/kg, *p* < 0.001, d = 0.57; Figure 4G), and 68%–76% (mean difference = 0.34 Nm/kg, *p* = 0.014, *d* = 0.49; Figure 4G) of the stance phase, but exhibited reduced hip external rotation moments during the terminal stance phase 88%–100% (mean difference = 0.83 Nm/kg, *p* = 0.002, *d* = 0.64; Figure 4G); (b) showed significantly greater knee abduction moments at 8%–12% (mean difference = 1.18 Nm/kg, *p* = 0.002, *d* = 0.63; Figure 4H) and 24%–92% (mean difference = 0.59 Nm/kg, *p* < 0.001, *d* = 0.67; Figure 4H) of the stance phase.

**Figure 5:**
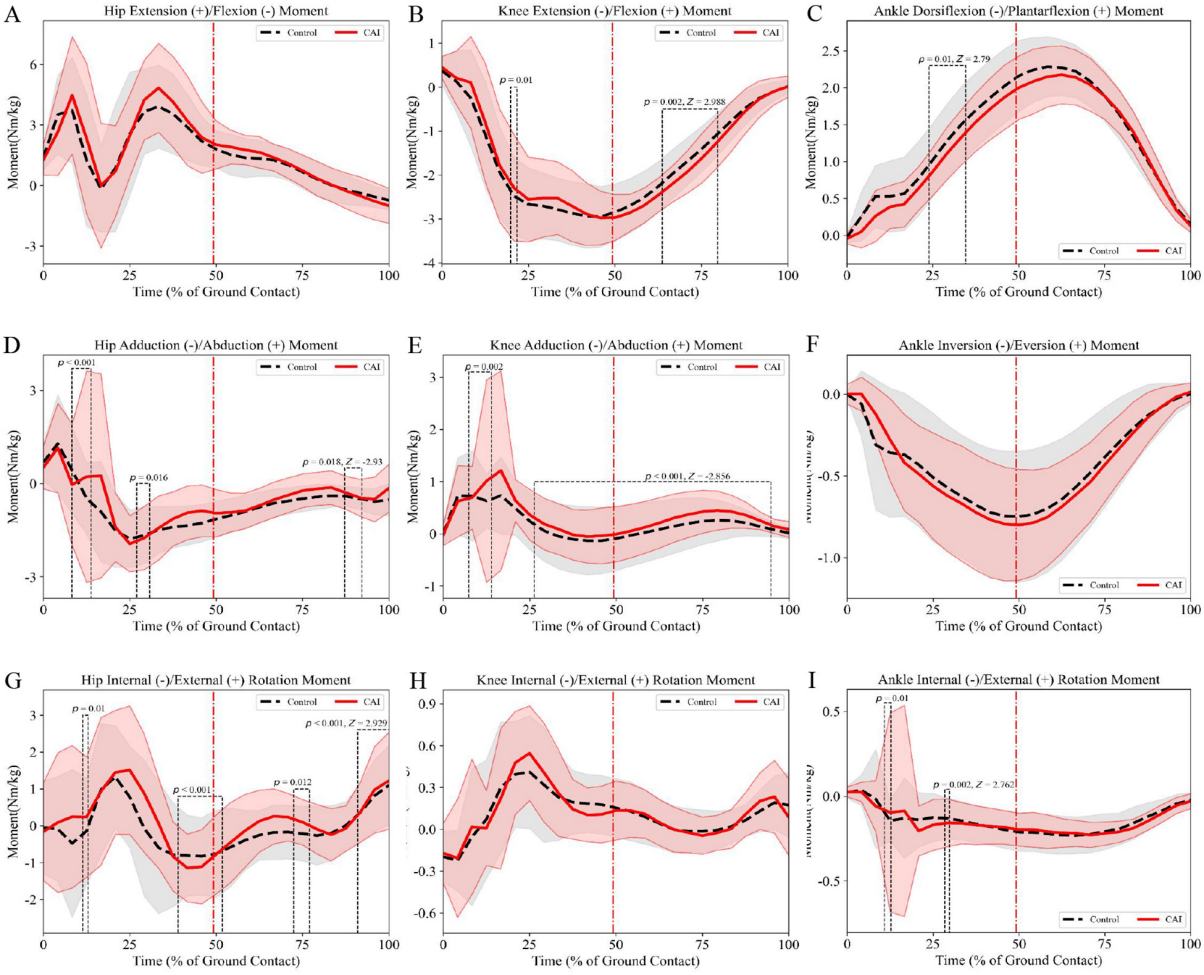
Female lower limb joint moments (mean ± SD) during side-cutting task: **(A–C)** sagittal plane moments, **(D–F)** frontal plane moments, **(G–I)**. Transverse plane moments. The red dotted line indicates the moment of maximum knee flexion during the maneuver.

Compared with the control group, frontal plane analysis demonstrated that female patients with CAI exhibited significantly greater knee abduction moments during 8%–12% (mean difference = 0.03 Nm/kg, *p* = 0.002, *d* = 0.04; Figure 5E) and 24%–92% (mean difference = 0.13 Nm/kg, *p* < 0.001, *d* = 0.27; Figure 5E) of the stance phase. Transverse plane analysis revealed that female patients with CAI displayed reduced hip external rotation moments at 8%–12% (mean difference = 0.72 Nm/kg, *p* = 0.01, *d* = 0.37; Figure 5G), 68%–72% (mean difference = 0.40 Nm/kg, *p* = 0.012, *d* = 0.59; Figure 5G), and 88%–100% (mean difference = 0.08 Nm/kg, *p* < 0.001, *d* = 0.09; Figure 5G) of the stance phase, but showed increased hip external rotation moments during 36%–48% of the stance phase (mean difference = 0.22 Nm/kg, *p* < 0.001, *d* = 0.21; Figure 5G).

No significant differences were observed in sagittal plane moments between CAI patients and controls (Figures 4A–C, 5A–C).

## 4 Discussion

This study conducted the first systematic investigation into the alterations of lower extremity joint kinematics and kinetics during side-cutting task in patients with chronic ankle instability. Incorporating sex-specific discussion and analysis, it reinforces previous findings regarding modified movement patterns in CAI patients. The primary findings revealed that CAI patients exhibited compensatory multi-plane angular adjustments at the hip, knee, and ankle joints, with direction and magnitude demonstrating significant sex disparities. Male CAI patients displayed greater hip flexion and external rotation angles, increased knee internal rotation angle, reduced knee flexion angle and ankle inversion angle compared to the healthy control group, along with enhanced hip external rotation moment and knee abduction moment. Female CAI patients manifested smaller hip and knee flexion angles, greater hip external rotation angle, increased ankle inversion and internal rotation angles relative to controls, coupled with diminished hip external rotation moment and elevated knee abduction moment.

### 4.1 Differences in lower limb joint kinematics

**In the sagittal plane**, male CAI patients exhibited greater hip flexion angles during side-cutting task, whereas the opposite was true for female CAI patients. Koshino et al. (17, 18) similarly found that CAI patients had greater hip flexion angles during side-cutting task than healthy controls. Normal individuals usually maintain balance by adjusting the center of gravity through an ankle strategy, in which the center of gravity rotates around the ankle, in the face of small, slow postural changes, whereas they usually use a hip strategy, in which the center of gravity is adjusted through hip flexion and extension, in the face of larger, faster postural changes (36). The side-cutting task usually requires the supporting foot to undergo rapid deceleration and transition to stirrup release, and the body requires faster faster postural changes; therefore, the increase in hip flexion may be a result of the hip balancing strategy (36). Hemami et al. (37) found that under translational platform disturbance, when the ankle joint is unable to generate sufficient torque, individuals must employ a hip strategy to maintain balance instead of an ankle strategy (36). This hip strategy may be a protective mechanism, as it has been shown that increased hip flexion during landing effectively reduces hip joint stiffness (24), which may help to eliminate the effects of ground reaction forces for a longer period of time, thus reducing the risk of lower limb injury (38). However, our results found a smaller hip flexion angle in female CAI patients compared to healthy controls, which may result in different movement patterns and joint angles due to differences in hip structure (39), strength and control between males and females (40). The hip is in a more extended position, which is not conducive to the absorption and dissipation of ground reaction forces, resulting in greater forces being transmitted to the knee, increasing the risk of knee injury. Additionally, a smaller hip flexion angle at landing increases the level of activation of the quadriceps muscles, which increases tibial forward shear, leading to an increased risk of anterior cruciate ligament (ACL) injury (41).

In the knee joint data, patients with CAI exhibited smaller knee flexion angles during the side-cutting task. Koshino et al. (13) found that patients with CAI exhibited greater knee flexion angles during the side-cutting task, which is contrary to our findings. This may be due to the fact that the side-cutting task required in the experiment of Koshino et al. (13) was a forward jump with a single-leg landing followed by a 45° side-cut, which emphasizes absorption of vertical impact forces and transmission of the kinetic chain in the sagittal plane. Deceleration is primarily achieved through modulation of lower limb stiffness during landing. In jump-side-cutting task, energy absorption relies more on progressive cushioning by the knee and hip joints, manifesting as greater knee flexion angles. However, this study required participants to perform a 5–10 meter sprint followed by active dissipation of horizontal kinetic energy while incorporating a side-cutting task. This “sprint-deceleration-side-cutting” composite task significantly increased joint loading in the frontal and transverse planes, which was more challenging for the CAI patients and better reflected the changes in proximal joint movement strategies due to the ankle joint instability. A decrease in knee flexion angle reduces the dissipation of energy by the muscle tissues surrounding the knee joint, resulting in a lack of energy attenuation capacity of the knee joint, which will be subjected to greater impact forces during landing, which may lead to knee cartilage damage (42). Decreased knee flexion angle during deceleration and increased contraction of the quadriceps muscles exerts a greater forward force, resulting in increased tibial shear, which the ACL and hamstrings are unable to resist, increasing the risk of ACL injury (43).

In the ankle joint data, we did not find any difference in the sagittal plane angle of the ankle between the two groups in the side-cutting task. This is consistent with the findings of Koshino and Terada et al. (13, 44). However, the study by Hopkins et al. (45) found that patients with CAI exhibited six distinct motor control strategies, suggesting that intragroup demographic differences may also influence experimental outcomes.

**In the frontal plane**, although our results found differences in hip and knee adduction and abduction angles between CAI patients and healthy controls in individual stance phases of the side-cutting task, these phases were not representative. When analyzed as a whole, no differences in hip and knee frontal plane angle were found between CAI patients and healthy controls during the side-cutting task. This is consistent with the findings of Herb et al. (44), but Koshino et al. (13) found that patients with CAI exhibited greater hip abduction angles during side-cutting task. Increased hip abduction prolongs the moment arm of the lower limb in the frontal plane, causing the ankle joint of the supporting leg to shift laterally. This enlarges the horizontal distance between the ankle joint and the projection line of the body's center of mass, thereby positioning the center of mass closer to the center of the base of support. Such adjustments enhance lateral stability and reduce ankle inversion during single-leg support under dynamic impacts or imbalances (46). Additionally, weakened hip abduction strength is a risk factor for ankle sprains in CAI patients (23, 47, 48). The increased hip abduction angle shortens the moment arm of the gluteus medius, allowing it to maintain frontal plane stability through low-threshold activation, thereby reducing the risk of injury (49).

In the ankle joint data, male CAI patients exhibited smaller ankle inversion angles around the moment of maximum horizontal backward ground reaction force in side-cutting task, while the opposite was true for females. The smaller ankle inversion angle in males suggests a more effective shock-absorbing strategy (50). The greater ankle inversion angle in females reflects the inability of the ankle to effectively absorb and dissipate ground reaction forces (44) and places the ankle in a position that is more susceptible to external ankle sprains, increasing the risk of re-sprains of the ankle, consistent with the findings of Koshino et al. (14).

**In the transverse plane**, patients with CAI, regardless of sex, exhibited greater hip external rotation during side-cutting task, which are inconsistent with the results of most current studies, which did not find a difference in hip cross-sectional angles between patients with CAI and healthy controls during side-cutting task (17, 18, 23), and the reasons why patients with CAI exhibit greater hip external rotation are not fully understood. It is not entirely clear why patients with CAI exhibit a greater hip external rotation angle. In the ACL injury literature, greater hip flexion is usually associated with greater hip external rotation or a landing posture linked to a lower risk of injury (51, 52). Since male CAI patients exhibit greater hip flexion, external hip rotation may be a corollary. And reduced hip external rotation angle is a risk factor for noncontact knee injuries because it can cause knee valgus.

In the knee joint data, male CAI patients exhibited greater knee internal rotation angles during the side-cutting task, whereas this was not found in females. Koshino et al. (14) did not find any differences in knee cross-sectional angles between CAI patients and healthy controls during the side-cutting task, which may be related to the fact that the side-cutting task we prescribed was more challenging for CAI patients. cutting maneuver is more challenging for CAI patients. It has been shown that an increased knee internal rotation angle places higher tension on the ACL and increases the risk of ACL injury (53).

In the ankle joint data, female CAI patients exhibited greater ankle internal rotation angles around the moment of maximum horizontal backward ground reaction force in the side-cutting task, which similar to the findings of Simpson et al. (54). This may have been demonstrated in conjunction with an increased ankle inversion angle, where the ankle inversion angle increases, the talonavicular tilt angle increases, and the talus is in a more internally rotated position (55). Additionally, existing studies have shown that individuals with CAI exhibit reduced preparatory muscle activity in the tibialis anterior. Insufficient contraction of the tibialis anterior fails to stabilize the talonavicular joint, leading to increased ankle internal rotation (56). Elevated ankle internal rotation angles amplify stress on the lateral joint space, and increased anterolateral pressure has been identified as one of the primary sources of pain in CAI (24). Beyond this, The increased angle of ankle internal rotation may be associated with coupling between ankle and hip external rotation (57).

### 4.2 Differences in lower limb joint kinetic

**In the sagittal plane**, although our results found differences in hip, knee and ankle moments between CAI patients and healthy controls in flexion (plantarflexion) and extension (dorsiflexion) moments in the individual stance phases of the side-cutting task, these phases were not representative. When analyzed as a whole, there were no differences in the sagittal moments of the hip, knee and ankle between CAI patients and healthy controls in the side-cutting task. In contrast, Simpson et al. (23) found that CAI patients exhibited greater ankle plantarflexion moments during a 45° side-cutting running maneuver after a forward jump landing, and that the increased plantarflexion moment may be a compensatory mechanism to overcome a more inwardly rotated ankle position at landing and to increase propulsive force for lateral change of direction (58). Heebner et al. (59) compared five commonly used landing maneuvers for biomechanical assessment and found that different landing actions elicited distinct biomechanical responses. Therefore, Our results are inconsistent with those of Simpson and Herb et al. (23, 44), which may be related to differences in the testing maneuver as well as the distance of the platform from the force plate.

**In the frontal plane**, although our results found differences in hip and ankle moments between CAI patients and healthy controls in adduction (inversion) and abduction (eversion) in the individual stance phases of the side-cutting task, these phases were not representative. When analyzed as a whole, there were no differences in the frontal moments of the hip and ankle between CAI patients and healthy controls in the side-cutting task. In contrast, Simpson et al. (23) found that CAI patients exhibited greater ankle plantarflexion moments and smaller eversion moments during a 45° side-cutting running maneuver after a forward jump landing. The decrease in ankle eversion moment suggests that when the lateral ankle contact with the ground is impacted, the lateral ankle musculature is unable to control the frontal plane centrifugal motion, resulting in excessive inversion of the ankle and an increased risk of re-spraining the ankle (23).

In the knee joint data, patients with CAI exhibit greater knee abduction moments during the side-cutting task. Simpson et al. (40) found that patients with CAI exhibited smaller knee abduction moments during a 45° side-cutting running maneuver after a forward jump landing, which is contrary to our findings and may be related to the fact that we prescribed a side-cutting task in the presence of rapid deceleration. Research suggests that the greater knee abduction moment in CAI patients may represent an attempt by the kinematic system to utilize the proximal segments of the kinetic chain to reduce loading and strain on the lateral structures of the ankle (11), but that an increased knee abduction moment increases the risk of ACL injury (53).

**In the transverse plane**, male CAI patients exhibited greater hip external rotation moments around the moment of maximum knee flexion angle during side-cutting task, whereas the opposite was true for females. Increased hip external rotation moment may be associated with increased knee internal rotation, which increases knee forces and instability and increases the risk of knee injury (40). However, the opposite results observed in females compared to males may be attributed to their relatively wider pelvis and greater anterior pelvic tilt during movement, which restricts the range of motion of the hip joint, thereby influencing hip joint moment patterns (39). Additionally, females exhibit lower pre-activation levels of neuromuscular activity in the hamstring muscles during side-cutting task. Such differences in neuromuscular coordination may reduce the control efficiency of the hip joint, ultimately limiting the output of external rotation moments (20).

In the knee joint data, although we found differences in rotational moments between the knees of CAI patients and healthy controls in some stance phases of the side-cutting task, these findings are not representative. When analyzed together, there were no significant differences in transverse moments between CAI patients and healthy controls in the knee joint during the side-cutting task. This is consistent with the findings of Simpson et al. (40).

Finally, this study reveals that patients with CAI exhibit significant proximal joint compensatory patterns and sex-specific biomechanical differences during dynamic side-cutting task, providing critical evidence for clinical assessment and rehabilitation strategies. In clinical practice, comprehensive evaluation of hip and knee joint movement patterns should be emphasized rather than focusing solely on the ankle joint. For male CAI patients, interventions should prioritize addressing excessive hip external rotation and abnormal knee joint moments through targeted training programs enhancing proximal joint dynamic stability. Female patients require focused attention on insufficient hip/knee flexion and imbalanced torque distribution, with neuromuscular control training to optimize lower limb kinetic chain efficiency. Future research should investigate long-term biomechanical consequences of compensatory patterns and the intrinsic mechanisms underlying sex differences, while employing multimodal assessments (e.g., EMG, kinematic−kinetic coupling analysis) to define triggering thresholds for proximal compensations. Additionally, developing personalized interventions targeting proximal joints (e.g., real−time biofeedback training) may effectively disrupt aberrant load transfer along the ankle-knee-hip chain, thereby reducing secondary injury risks from chronic structural overload and advancing theoretical frameworks for precision rehabilitation in CAI.

### 4.3 Limitations and perspectives

Although our findings support our research hypothesis, limitations of the current study should be considered when interpreting the results. First, the retrospective study design could not elucidate when the lower extremity biomechanical changes in patients with CAI appeared; it is possible that these changes existed before the ankle sprain, and future longitudinal follow-up studies should be conducted to address this issue. Second, this study only included patients with CAI and healthy individuals without CAI, considering that there are also individuals with a history of ankle sprains without residual symptoms or deficits who are able to return to a high level of activity after sustained acute lateral ankle sprains without any residual injury, biomechanical changes in the lower extremity of this group of individuals should be investigated in future studies that will help us to better understand the CAI-occurring mechanisms of CAI occurrence. Third, the test movements in this study were anticipatory and artificial in the laboratory, which means that subjects were focused on the task at hand, potentially masking possible kinematic differences between patients with CAI and healthy controls, and it is debatable to what extent they reflect actual competition and training situations. Future studies should consider using an unintended test task to further understand the kinematics and neuromuscular control of the lower extremity in patients with CAI. Fourth, although skin-reflective markers are commonly used to assess joint kinematics, they are limited by soft-tissue artifacts that can lead to errors in lower-extremity kinematic data, particularly in the frontal plane and cross-sections. We minimized errors associated with skin reflective markers by using the following steps: reflective markers were applied by the same experimenter in this study to increase the reliability of marker placement and to avoid potential errors caused by inter-tester variability. Fifth, our study did not analyze changes in trunk kinematics and kinetics. Landing and jumping tasks require trunk musculature to stabilize the trunk against backward ground reaction forces at the moment of touchdown. Like the altered hip kinematics we observed, altered trunk flexion may be a compensatory strategy used by CAI patients during landing and jumping. Sixth, we did not clearly elucidate how foot motion in patients with CAI affects knee or hip motion because we defined the foot as a whole, but in reality the foot is composed of multiple joints. Finally, we did not directly examine ACL loading. We interpreted these results in light of previously proposed mechanisms for increasing or decreasing ACL load. Therefore, further research to directly estimate the relationship between ACL strain or load and lower extremity joint biomechanics could lead to more specific conclusions.

## 5 Conclusion

**Figure 6:**
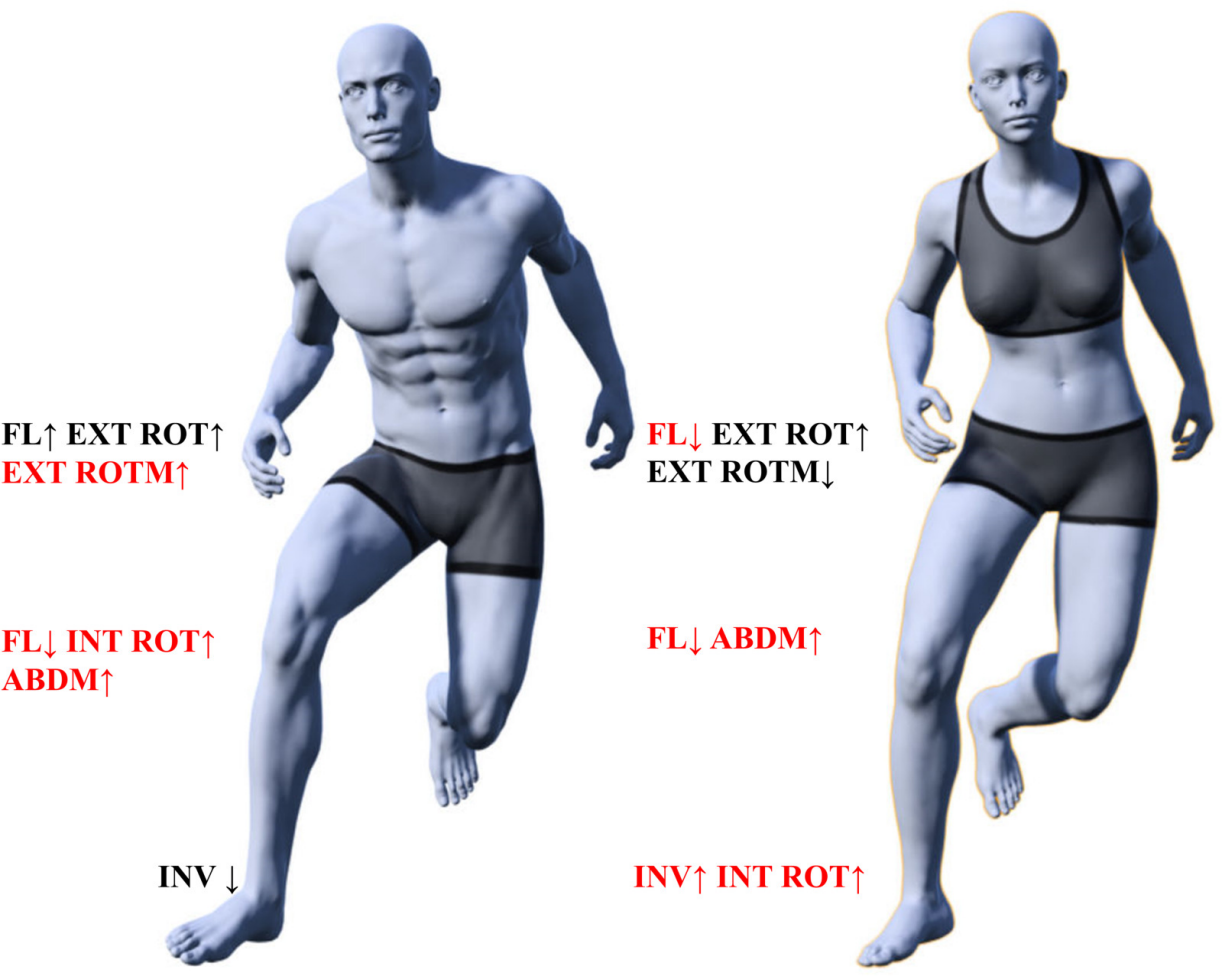
Lower extremity biomechanics of CAI patients of different sexes during side-cutting task. ABDM, abduction moment; EXT ROT, external rotation angle; EXT ROTM, external rotation moment; FL, flexion angle; INV, inversion angle; INT ROT, internal rotation angle, red font indicates the unfavorable factors of lower limb injury.

Our data analysis showed that during the side-cutting task (Figure 6), male CAI patients exhibited greater hip flexion and external rotation, greater knee internal rotation, smaller knee flexion and ankle internal rotation, and greater hip external rotation and knee abduction moments compared to healthy controls; female CAI patients exhibited smaller hip and knee flexion angles, greater hip external rotation and ankle internal rotation, and smaller hip external rotation moments and greater knee abduction moments compared to healthy controls. Female CAI patients showed smaller hip and knee flexion angles, larger hip external rotation and ankle internal rotation angles, smaller hip external rotation moments, and larger knee abduction moments than healthy controls.

In summary, patients with CAI exhibit altered movement patterns in the hip, knee, and ankle joints across multi-planar angles, with significant sex-based disparities in both direction and magnitude. Male patients demonstrate a generalized increase in range of motion and torque at proximal joints, whereas female patients display reduced proximal joint motion but elevated knee abduction torque. Notably, increased knee abduction torque is observed in both male and female CAI groups, which may reflect adaptive adjustments in load distribution across planes other than the sagittal plane at the knee joint. Furthermore, the greater hip external rotation moment and greater knee internal rotation angle demonstrated by male CAI patients, the smaller hip flexion angle and greater ankle internal rotation angle demonstrated by female CAI patients, and the smaller knee flexion angle and greater knee abduction moment common to both sexes may impair the ability of the lower limb joints to absorb and dissipate ground reaction forces, thereby increasing contact stress, shear force, or compressive force on static stabilizing structures, which could elevate the risk of lower limb injuries.

## Data Availability

The original contributions presented in the study are included in the article/Supplementary Material, further inquiries can be directed to the corresponding authors.
